# Primary renal angiosarcoma with progressive clinical course despite surgical and adjuvant treatment: A case report

**DOI:** 10.3892/ol.2015.2902

**Published:** 2015-01-27

**Authors:** FILIZ CELEBI, KEZBAN NUR PILANCI, SEZER SAGLAM, NUMAN CEM BALCI

**Affiliations:** 1 Department of Radiology, Gayrettepe Florence Nightingale Hospital, Istanbul 34340, Turkey; 2 Department of Oncology, Istanbul Bilim University, Istanbul 34340, Turkey

**Keywords:** renal angiosarcoma, nephrectomy, cluster of differentiation 31, cluster of differentiation 34

## Abstract

Angiosarcoma is an extremely rare, high-grade malignancy, which accounts for <2% of all soft-tissue sarcomas. Cases of primary renal angiosarcoma represent 1% of these. Angiosarcomas involving the kidney usually originate from metastatic skin lesions or primary visceral lesions and most often occur in the sixth and seventh decades of life. The present study describes a case of primary renal angiosarcoma that presented as a large right-sided renal mass with symptoms of flank pain. Despite surgical removal of the tumor, recurrent disease with associated lung metastases was identified at the surgical site following adjuvant chemotherapy. The patient succumbed to the disease 13 months after the diagnosis.

## Introduction

Angiosarcoma is a rare malignant neoplasm that affects the endothelial-type cells lining the vessel walls (1). Overall, it accounts for <2% of all soft-tissue sarcomas. Since the first report of the disease in 1942 (2), extremely few cases have been described in PubMed. Visceral sarcomas are less common than soft-tissue and skin sarcomas, and renal involvement is generally associated with metastasis (3). Primary renal angiosarcoma usually affects Caucasians in the sixth and seventh decades of life. In general, it presents with macroscopic hematuria (81%), pain in the flank (38%) or a palpable renal mass (31%) (4). Here, we present the case of a patient with primary renal angiosarcoma with pleuropulmonary metastasis. The aim of this report is to provide additional information regarding imaging techniques and the clinical behaviour of angiosarcoma to aid with its differential diagnosis. Written informed consent was obtained from the patient’s family.

## Case report

A 57-year-old male was referred to the Gayrettepe Florence Nightingale Hospital (Istanbul, Turkey) with right-sided flank pain and hematuria. A physical examination revealed tenderness in the right hypochondrium, with otherwise normal clinical findings. The routine blood tests revealed anemia with a hemoglobin level of 10.5 g/dl (normal range, 13–17 g/dl). The patient underwent abdominal sonography, which identified a large right-sided renal mass and a simple cortical cyst in the left kidney. The remaining blood tests and tumor marker levels [carcinoembryonic antigen, cancer antigen (CA)19-9 and CA-125] were normal. There was no history of exposure to radiation, vinyl chloride or thorium dioxide (also known as thorotrast).

**Figure 1:**
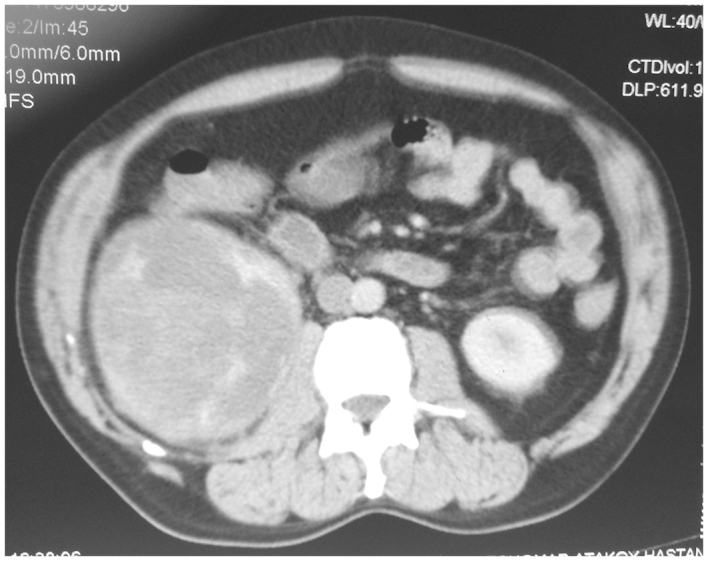
Contrast-enhanced computed tomography revealing a centrally necrotized and hemorrhagic right-sided renal mass with heterogenous peripheral enhancement.

Computed tomography (CT) identified a renal mass measuring 14×12 cm. The lesion demonstrated heterogenous peripheral enhancement with central areas of necrosis and hemorrhage (Fig. 1). Radiological differential diagnosis concluded that the lesion was most likely renal cell carcinoma or a metastasis from another primary tumor. The mass was surgically removed by radical nephrectomy. The surgical specimen consisted of the entire right kidney, which weighed 1,080 g and measured 18×13×10 cm, with a 14×12×9 cm mass that occupied the majority of the tissue. The tumor was composed of spindle and epithelioid cells with high-grade morphologies, and large, necrotic hemorrhagic areas. Within the tumor, five mitoses per 10 high-power fields were also observed. Normal renal tissue, measuring 7×5×4 cm, was evident on one side of the resected specimen. There was no evidence of thrombosis in the renal vein. Immunohistochemical findings revealed that the neoplastic cells were highly positive for cluster of differentiation (CD)31 and CD34, which supported the diagnosis of primary renal angiosarcoma.

**Figure 2:**
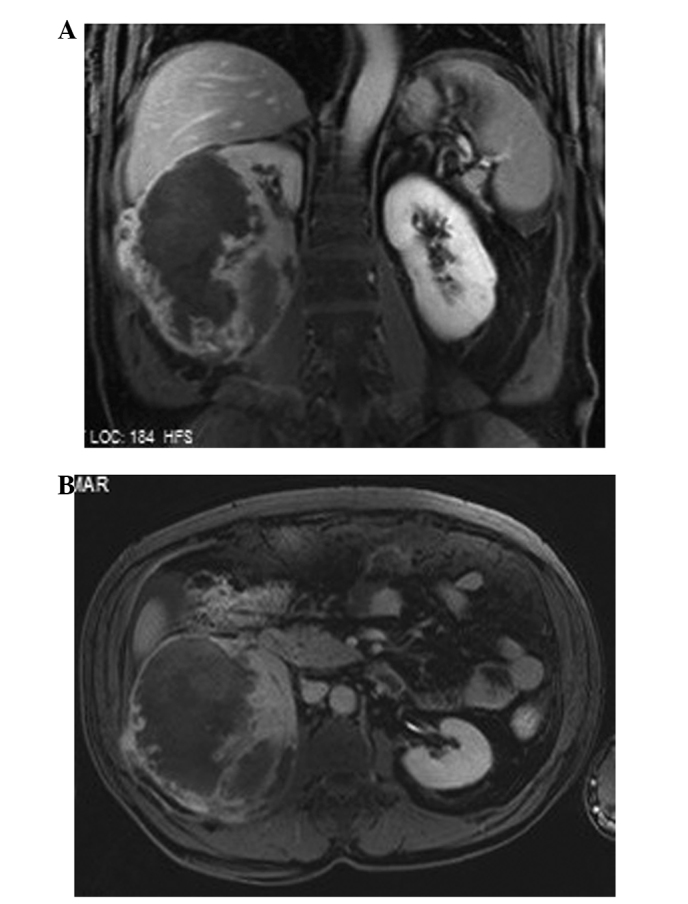
Contrast-enhanced three-dimensional magnetic resonance imaging in (A) coronal and (B) axial planes revealing a necrotic mass in the lower portion of the right kidney with no central enhancement.

**Figure 3:**
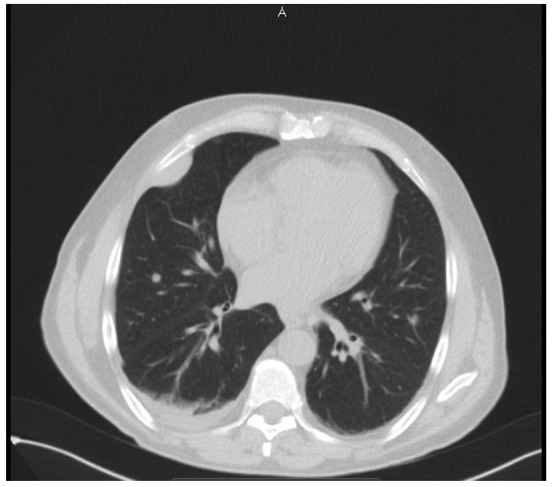
Computed tomography image of the thorax revealing the presence of multiple pleuropulmonary metastatic nodules and pleural effusion.

Subsequent to diagnosis, two cycles (each cycle lasts two weeks) of the tyrosine kinase inhibitor, sunitinib, were administered at a dose of 37.5 mg/day. A few months later, the patient was referred to the Gayrettepe Florence Nightingale Hospital with recurrence of the right-sided flank pain. Subsequent to a physical examination, abdominal magnetic resonance imaging (MRI) and chest CT were performed. The abdominal MRI scan revealed a heterogenous contrast-enhanced necrotic mass measuring 8×9.5×12.5 cm (Fig. 2) in the right nephrectomy space, and a 6.5×11 cm pelvic metastatic mass in the iliac fossa. CT images of the thorax identified multiple pleuropulmonary metastatic nodules (Fig. 3). As no standard chemotherapy protocol exists for the treatment of renal angiosarcomas, two weeks of palliative chemotherapy was initiated with 85 mg/m^2^ oxaliplatin and 130 mg/m^2^ paclitaxel (5). In total, four cycles of chemotherapy were administered. Despite treatment, there was no radiological response, and only pain palliation was achieved. Following six cycles of chemotherapy, the re-evaluation abdominal MRI and chest CT revealed pleuropulmonary progression and no change in the size of the right recurrent mass or pelvic mass. Due to the progressive nature of the disease, chemotherapy with oxaliplatin and paclitaxel was ceased. Bevacizumab (7.5 mg/kg) was then administered every three weeks. However, there was no response to three cycles of the antiangiogenic therapy and the patient succumbed to the disease 13 months after the initial diagnosis.

## Discussion

Renal angiosarcomas are extremely rare neoplasms that originate from the normal endothelium. Overall, the lesions account for ~1% of all soft-tissue sarcomas (3–7). In total, <30 cases of renal angiosarcoma have been reported since 1942, and at present, the etiology of the disease remains unknown. The risk factors for renal angiosarcomas are uncertain, but may include renal transplantation, post-treatment lymphedema, and exposure to arsenic, vinyl chloride, thorotrast and radiation (3,8,9).

Early diagnosis is challenging, as the associated clinical symptoms do not develop in the early stages of tumor growth, and the disease often progresses rapidly (10). Metastases are often present at the time of diagnosis or develop due to hematogenous spread within a couple of months or weeks after surgery. The common metastatic sites are the lungs, liver and bones (8,11,12). Overall, an extremely small number of patients survive more than one year from the time of diagnosis. Clinical and radiological findings can only identify the presence of a malignant tumor; the definite diagnosis depends upon the histopathological examination of the nephrectomy specimen and positive endothelial markers with immunohistochemical staining. The most commonly used markers are CD31, an endothelial cell adhesion molecule with high specificity and sensitivity, and CD34, a human hematopoietic progenitor cell antigen (3,12).

The prognosis of a patient with renal angiosarcoma depends upon the size of the initial lesion and the presence or absence of metastasis at diagnosis. Tumors measuring <5 cm in diameter confer an improved prognosis compared with those >5 cm (11,12). The five-year survival rates for tumors >5 and <5 cm are 13 and 32%, respectively (9).

Local recurrence following surgery is common for angiosarcomas. In the majority of cases, metastasis is present prior to the time of diagnosis. Due to the rarity of the malignancy, it is challenging to establish a standardized treatment protocol for the local disease. Radical surgery with adjuvant chemotherapy is, at present, considered to be the optimum treatment option for angiosarcomas (9).

In conclusion, in this study the rare case of primary renal angiosarcoma with large necrotic areas was presented, which was firstly misdiagnosed radiologically and clinically as necrotic renal cell carcinoma. The present case highlights the importance of understanding the imaging features of this rare aggressive malignancy.
